# Validation of an Online Food Frequency Questionnaire against Doubly Labelled Water and 24 h Dietary Recalls in Pre-School Children

**DOI:** 10.3390/nu9010066

**Published:** 2017-01-13

**Authors:** Christine Delisle Nyström, Hanna Henriksson, Christina Alexandrou, Anna Bergström, Stephanie Bonn, Katarina Bälter, Marie Löf

**Affiliations:** 1 Department of Biosciences and Nutrition, Karolinska Institutet, NOVUM, 14183 Huddinge, Sweden; alexandrou81@gmail.com (C.A.); marie.lof@ki.se (M.L.); 2 PROFITH “Promoting FITness and Health through Physical Activity” Research Group, Department of Physical Education and Sports, Faculty of Sports Sciences, University of Granada, 18071 Granada, Spain; hannahenriksson77@hotmail.se; 3 Department of Environmental Medicine, Karolinska Institutet, 17177 Stockholm, Sweden; anna.bergstrom@ki.se; 4 Department of Medical Epidemiology and Biostatistics, Karolinska Institutet, 17177 Stockholm, Sweden; stephanie.bonn@ki.se (S.B.); katarina.balter@ki.se (K.B.)

**Keywords:** doubly labelled water, food frequency questionnaire, 24 h dietary recalls, pre-school

## Abstract

The development of easy-to-use and accurate methods to assess the intake of energy, foods and nutrients in pre-school children is needed. KidMeal-Q is an online food frequency questionnaire developed for the LifeGene prospective cohort study in Sweden. The aims of this study were to compare: (i) energy intake (EI) obtained using KidMeal-Q to total energy expenditure (TEE) measured via doubly labelled water and (ii) the intake of certain foods measured using KidMeal-Q to intakes acquired by means of 24 h dietary recalls in 38 children aged 5.5 years. The mean EI calculated using KidMeal-Q was statistically different (*p* < 0.001) from TEE (4670 ± 1430 kJ/24 h and 6070 ± 690 kJ/24 h, respectively). Significant correlations were observed for vegetables, fruit juice and candy between KidMeal-Q and 24 h dietary recalls. Only sweetened beverage consumption was significantly different in mean intake (*p* < 0.001), as measured by KidMeal-Q and 24 h dietary recalls. In conclusion, KidMeal-Q had a relatively short answering time and comparative validity to other food frequency questionnaires. However, its accuracy needs to be improved before it can be used in studies in pre-school children.

## 1. Introduction

Diet is just as important in children as in adults, and possibly even more so because the habits developed in the early years often persist throughout the lifespan [1]. Being able to measure diet in pre-school children is important since childhood obesity often continues into adulthood [2]. An unhealthy diet is a contributing risk factor for some of the most common non-communicable diseases, such as cardiovascular disease, diabetes and cancer [3]. In order to investigate the relationships between diet and disease it is imperative to be able to easily and accurately measure energy and food intake, especially in large epidemiological settings.

Traditional methods to assess energy and food intake are 24 h dietary recalls, dietary history, diet records and food frequency questionnaires (FFQ). However, these are burdensome for both the participants and researchers and their accuracy is limited [4]. Thus, the development of easy-to-use and accurate methods to assess the intake of energy, foods and nutrients in pre-school children are required. Over the past 10 years, there has been great interest in using telecommunications and computer technology to develop such methods [5]. The use of online questionnaires in epidemiology can be advantageous by allowing for faster response rates, reducing incomplete data via automated controls and increasing the ease of data processing through immediate storage of digital data [6,7].

The LifeGene prospective cohort study is an ongoing epidemiological study in Sweden, which will include approximately 300,000 people, aged 18 to 45. It aims to provide new information on various diseases and health issues affecting society such as cancer, cardiovascular disease, obesity and allergies [8]. LifeGene is currently assessing diet in adult participants using an online questionnaire, MiniMeal-Q. MiniMeal-Q has been validated against doubly labelled water (DLW) and has been shown to be valid for use in epidemiological studies [9]. As there are many differences in the types and amounts of food consumed, it is necessary to develop and validate online questionnaires for other age groups, such as pre-school children.

We included the FFQ KidMeal-Q in the population-based randomized controlled trial called Mobile-based intervention intended to stop obesity in pre-schoolers (MINISTOP). Overall, this trial aims to determine the effectiveness of a six-month mobile-phone-based intervention to improve body composition, dietary habits, physical activity, sedentary behaviour and physical fitness in healthy pre-school children [10]. The specific aims of this nested validation study are to compare: (i) energy intake (EI) obtained using KidMeal-Q to total energy expenditure (TEE) measured via DLW and (ii) the intake of certain foods measured using KidMeal-Q to intakes acquired by means of 24 h dietary recalls in 38 children aged 5.5 years.

## 2. Materials and Methods

### 2.1. Participants and Study Design

The MINISTOP trial was based in the county Östergötland in Sweden. A total of 40 parent couples and their children agreed to participate in a validation of dietary intake [11], body composition [12], and physical activity methods at the final follow-up assessment, which began in February 2015 when their children were 5.5 years of age. Details of the recruitment and population have been published previously [11,12]. The age, weight, height, body mass index (BMI), as well as the parental age, BMI and education were comparable between this sample and those in the whole MINISTOP trial (*n* = 315). Two children had missing information and in total 38 (18 from the intervention group and 20 from the control group) 5.5-year-olds participated in this validation.

This study was conducted according to the guidelines laid down by the Declaration of Helsinki, all procedures involving human subjects were approved by the Research and Ethics Committee in Stockholm, Sweden (2013/1607-31/5; 2013/2250-32), and informed consent was obtained from all parents. The MINISTOP trial is registered as a clinical trial (https://clinicaltrials.gov/ct2/show/NCT02021786).

### 2.2. Protocol

Parents of the children collected two urine samples at home and brought them to the measurement session at the Linköping University Hospital. The weight and height of the children were recorded when they were wearing minimal clothing and no shoes. Thereafter, the child received a dose of stable isotopes mixed with fruit juice to measure their TEE during the subsequent two-week period. The parents were instructed to collect urine samples on days 1, 5, 10 and 14 after dosing and to note the time of sampling. Within the same two-week period the intake of food and drink was assessed using 24 h dietary recalls. After the measurement at the hospital, all parents received an e-mail with a link to the online FFQ KidMeal-Q and were instructed to fill it in directly after the visit.

### 2.3. Energy Expenditure

TEE has been measured as previously described [11]. Briefly, each child was given an accurately weighed dose of stable isotopes, 0.14 g ^2^H_2_O and 0.35 g H_2_^18^O, per kg body weight. Five urine samples were collected (on days 1, 5, 7, 10 and 14), stored and analysed for isotope enrichments as previously described [11]. CO_2_ production was calculated according to Davies et al. [13], assuming that 27.1% of the total water losses was fractionated [13,14]. TEE was calculated by means of the Weir equation [15], assuming a food quotient of 0.85 [16]. The mean change in body weight from day one to 14 was 0.07 ± 0.32 kg.

### 2.4. KidMeal-Q

KidMeal-Q is an online meal-based FFQ designed for pre-school children aged three to six. This FFQ measures the child’s dietary intake over the past couple of months and includes between 42 and 86 food items, drinks and dishes, depending on the number of follow-up questions. The following pre-defined frequency categories were used: for breakfast food items as well as fruit (1 time/day, 2 times/day or more, 1–2 times/week, 3–6 times/week, 1–3 times/month), for dishes, snacks as well as sweets (1–2 times/week, 3–6 times/week, 7 times/week or more, 1–3 times/month), and for vegetables (1 time/day, 2 times/day, or 3 times a day or more). See the Supplementary Materials for the questions provided in KidMeal-Q. For each of the following food groups, five photos of portion sizes were included: (1) rice, potatoes and pasta; (2) meat, chicken, fish and vegetarian substitutes; and (3) vegetables (raw or cooked).The photos were used to calculate portion sizes for cooked dishes and vegetables. For other food items, standard portions were used. EI was calculated from reported intakes of food items and dishes by linkage to the food composition database provided by the National Food Administration [17] by means of KidMealCalc (Epiqcenter, Stockholm, Sweden), a software developed and validated for this purpose. The grams of fruits, vegetables, fruit juice, sweetened beverages, candy, ice cream and bakery products were then summarized. These foods were selected as they represent healthy and unhealthy food habits relevant for childhood obesity [11].

### 2.5. 24 h Dietary Recalls

Four 24 h dietary recalls were performed over the phone in the two-week period following the measurement at the hospital, as published previously [11]. The days used in the 24 h dietary recalls were scheduled with the parents when they were at the hospital for measurements. Briefly, each parent was asked to recall the foods and beverages their child consumed. Information on the type of food products used in mixed dishes and the cooking method was recorded. The portion sizes were reported by the parents using household measurements (decilitres, tablespoons or teaspoons). Words such as slices or pieces were used for other foods such as bread, candy or potatoes. The reported intakes were converted into grams using a standardized weight table provided by the Swedish Food Agency [18] and the grams of fruits, vegetables, fruit juice, sweetened beverages, candy, ice cream and bakery products were then summarized. EI and nutrients were calculated from reported intakes of foods and beverages by linkage to the food composition database [17].

### 2.6. Statistics

Values are given as means and standard deviations (SD). Significant differences between mean values were identified using paired samples *t*-tests and the Wilcoxon Signed Rank test for parametric data (EI, TEE and selected nutrients) and non-parametric data (food groups). Pearson or Spearman correlations were used to evaluate relationships between variables. The Bland and Altman procedure [19] was used to compare EI using KidMeal-Q to TEE measured via DLW. Thus, the difference (*y*) between EI and TEE was plotted versus the average of the two estimates (*x*). The mean difference with ±2SD (limits of agreement) were then calculated. To test for a relationship between *x* and *y* in the Bland and Altman plot, linear regression was used. Significance (two-sided) was accepted when *p* < 0.05. Analyses were performed using SPSS version 23 (IBM, Armonk, NY, USA).

The classification capacity of KidMeal-Q was assessed using TEE. This was done by ranking EI (KidMeal-Q) and TEE (DLW) in a sequence. Thus, the children with the lowest EI and TEE had the lowest number and the difference between this child and the second in the sequence was the smallest possible. This principle of the smallest possible difference was maintained for all children, producing a sequence with gradually increasing values. The children were then divided into tertiles (low, medium and high) with increasing values. The classification capacity for KidMeal-Q was then evaluated as the number of children placed in the same (0), in the next higher (+1) or lower (−1) and in the second next higher (+2) or lower (−2) group.

## 3. Results

The descriptive characteristics and the energy expenditure for the 38 children (22 boys and 16 girls) are displayed in Table 1. There were no significant differences in anthropometric measures between boys and girls and therefore all analyses are presented for boys and girls combined. There was a wide range for weight and energy expenditure for the children. The parents were highly educated, with between 65%–75% of the parents having a university degree.

**Figure 1:**
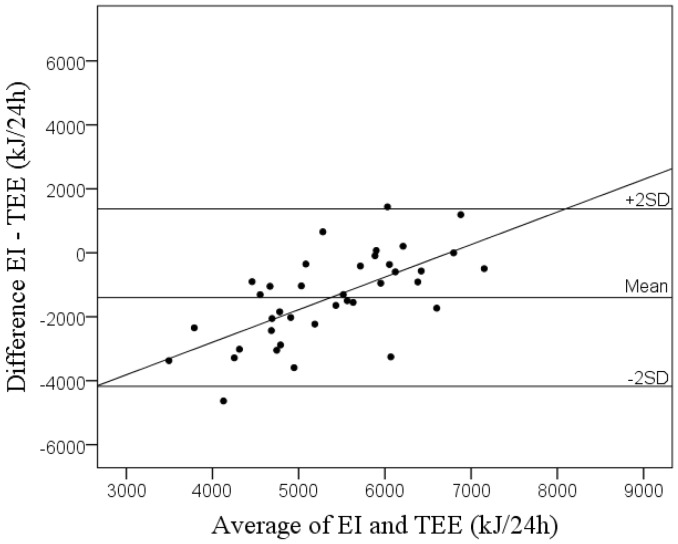
Bland and Altman Plot comparing energy intake using KidMeal-Q and total energy expenditure calculated using doubly labelled water in 38 children aged 5.5 years. The mean difference between methods was −1400 kJ/24 h with limits of agreement (2SD) of 2775 kJ/24 h.

On average it took the parents of the participating children 13.2 ± 6.2 min to complete KidMeal-Q. The mean EI calculated using KidMeal-Q was statistically different (*p* < 0.001) from TEE assessed via DLW. The mean EI was 4670 ± 1430 kJ/24 h and TEE was 6070 ± 690 kJ/24 h. Figure 1 displays the Bland and Altman Plot for EI assessed using KidMeal-Q to TEE measured using DLW. The limits of agreement were wide and a significant association was found for the average and difference (*r* = 0.711, *p* < 0.001). A significant trend was found, showing that lower EIs were underestimated to a greater extent. In comparison to TEE, KidMeal-Q underestimated EI in 84.2% (*n* = 32).

A significant correlation was found between EI (KidMeal-Q) and TEE (DLW), *r* = 0.320 (*p* = 0.05). When dividing the children into tertiles (low, medium and high) for EI and TEE 42.1% (*n* = 16) were classified correctly, 47.4% (*n* = 18) were classified plus or minus one group, and 10.5% (*n* = 4) were classified plus or minus two groups.

Table 2 shows the mean intakes and the correlations for the seven foods and drinks assessed using KidMeal-Q and 24 h dietary recalls. Only sweetened beverage consumption was significantly different in mean intake (*p* < 0.001) as measured by KidMeal-Q and 24 h dietary recalls. Significant correlations were observed for vegetables, fruit juice and candy between the two methodologies. Table 3 displays the mean intakes and correlations for selected nutrients estimated using KidMeal-Q and 24 h dietary recalls. For the percentage of energy obtained from the macronutrients no significant differences were observed, however a significant difference in percent energy from sucrose (*p* < 0.001) was found. Significant differences were also found for fibre and calcium (both *p* < 0.001). Significant correlations were found for the majority of the selected nutrients. EI from KidMeal-Q and the 24 h dietary recalls were correlated (*r* = 0.532, *p* = 0.001).

## 4. Discussion

KidMeal-Q is an interactive and user-friendly questionnaire with a relatively short answering time and has comparable validity to other corresponding epidemiological tools. KidMeal-Q underestimated EI in the majority of children. However, in regards to the seven investigated food groups only one significant difference was found (sweetened beverages) for the mean intakes assessed using KidMeal-Q and 24 h dietary recalls.

KidMeal-Q is quick and simple for parents to respond to, which increases user-friendliness and the likelihood of completion [20]. On average a FFQ takes between 30 to 60 min [21], while KidMeal-Q took only a quarter to a half of that time. A systematic review and meta-analysis conducted by Edwards et al. [20] found that response rates are inversely related to the questionnaire length, and found an even stronger relationship in extremely short questionnaires. Time is not the only factor that affects user-friendliness; the wording and layout of the questions also plays a large role [22]. The interactive design of KidMeal-Q allows for easy navigation throughout the questionnaire through prompts, error messages and letting participants skip irrelevant questions, all of which increases completion rates [6,23]. The short answering time and interactive design of the questionnaire allows it to be used on a large number of people as well as in epidemiological studies, where dietary habits are not part of the main research question and only play a small part in the study.

As found with other FFQs [9,24,25,26,27] KidMeal-Q had wide limits of agreement, demonstrating that it is not a valid tool on an individual level. In comparison with TEE from DLW KidMeal-Q underestimated EI by 23%. One FFQ in pre-school children overestimated EI in comparison to TEE from DLW by 59% [28] and two others underestimated EI by 3% [26] and 5% [25]. Similar to KidMeal-Q, two of the FFQs [26,28] were conducted under unsupervised conditions, whereas that by Collins et al. [25] was conducted in a supervised setting, possibly allowing for the questionnaire to better predict EI through allowing parents to ask questions and clarify statements. The more questions in the Dutman et al. FFQ are more detailed [26], as demonstrated by its longer answering time of 25 min, may have led to a more accurate reporting of EI intake in comparison to KidMeal-Q. KidMeal-Q differed from the aforementioned FFQ in terms of how the parents reported their child’s food intake, online versus pen and paper, which also may have led to the observed differences. The underestimation of EI could possibly be due to the questionnaire itself; for instance, the portion sizes provided were perhaps too small and thus led to the observed underestimation. It is important to note that KidMeal-Q underestimated sweetened beverages on average by 82 grams per day. This amount corresponds to approximately 1370 kJ, which is a considerable amount of energy. As FFQs have been shown to both under- and overestimate EI, more research needs to be conducted to improve the accuracy of these tools. Further work should focus on examining the provided portion sizes as well as gain additional understanding of parental reporting of dietary data. Specifically for KidMeal-Q, a revision of the questions regarding sweetened beverages is required.

Even though correlations are not optimal when evaluating methods, they are often used when comparing dietary assessment methods. In this study a significant moderate correlation was found between EI from KidMeal-Q and TEE measured with DLW, with similar results being found in other studies [9,24]. However, the correlation was lower than Kroke et al. [27] and Dutman et al. [26], but higher than Collins et al. [25] and Perks et al. [29]. Four of the six FFQs studied [9,24,27,29] were conducted in an adult or youth population and were traditional paper-based questionnaires, except for Christensen et al. [9]. A stronger correlation was found in the Dutman et al. [26] study; however, this may be attributed to the fact that they extensively reviewed their FFQ results and contacted parents about peculiar answers, which should provide more accurate estimates of EI. KidMeal-Q demonstrated a decent ranking ability compared to DLW, which is similar to other studies [9,26,27]. In regards to the correlations for the seven investigated food groups, they are also similar to those found in previous studies in pre-school children [30,31].

A strength of this validation study was the use of DLW as a reference method, which is considered the gold standard for assessing TEE and recommended for usage when validating EI [32]. Furthermore, the use of 24 h dietary recalls allowed us to assess KidMeal-Q’s ability to evaluate certain food groups, which is of great importance in epidemiological studies. This study was limited by the fact that TEE was the average of 14 days, while KidMeal-Q assessed dietary habits over the past couple of months; however, the day-to-day variation in TEE is low [33,34], thus we do not think this fact has largely influenced our results. We were unable to obtain four 24 h dietary recalls from all participants; however, when we re-ran the analyses including only children with four 24 h dietary recalls (*n* = 26), our conclusions remained the same. Furthermore, this nested validation study was conducted at the final follow-up within the MINISTOP trial and the parents in the intervention group were given advice on how to make their child’s diet more healthy, which could have affected how they answered the FFQ. We do not believe this is an issue as there were no significant differences in EI as measured by KidMeal-Q or 24 h dietary recalls, TEE, or the food groups between the children in the intervention and control group. Additionally, the majority of the 24 h dietary recalls were weekend days as the MINISTOP trial targeted the home environment. However, as we have stated previously [11], we do not believe this is a major issue because the majority of Swedish parents with a child this age work and would have their child in daycare, so when they filled out the FFQ they would more than likely be filling it out with their child’s food habits from the home environment. This study also had a relatively small sample size (*n* = 38) and only four 24 h dietary recalls were applied. Finally, the fact that the parents were on average more highly educated than the general Swedish population may limit the generalizability of the results.

In conclusion, the online FFQ, KidMeal-Q, has been demonstrated to be interactive and user-friendly. It has a relatively short answering time and has comparative validity to other FFQs. However, more work is needed to further improve the questionnaire’s accuracy before it can be used in studies in pre-school children.

## Figures and Tables

**Table 1:** Age, weight, height and energy expenditure of the study participants (*n* = 38).

Variables	Mean ± SD	Range
Age (years)	5.5 ± 0.1	5.2–5.7
Weight (kg)	20.6 ± 4.3	14.9–35.8
Height (cm)	114 ± 5	105–126
BMI (kg/m^2^)	15.6 ± 2.3	13.3–25.6
TEE (kJ/24 h)	6070 ± 690	4910–7700
EI (kJ/24 h) ^1,3^	4670 ± 1430	1810–7480
EI (kJ/24 h) ^2^	6025 ± 665	4515–7530

SD, standard deviation; BMI, body mass index; TEE, total energy expenditure measured via doubly labelled water; BW, body weight; EI, energy intake. ^1^ EI assessed using KidMeal-Q; ^2^ EI assessed using 24 h dietary recalls; ^3^ Significantly lower than TEE as well as EI from 24 h dietary recalls (both *p* < 0.001).

**Table 2:** Mean intakes and correlations for the selected foods and beverages estimated by KidMeal-Q and 24 h dietary recalls (*n* = 38).

	KidMeal-Q (g/Day)	24 h Dietary Recall (g/Day) ^1^	*p*-Value ^2^	Rho ^3^	*p*-Value ^4^
**Fruit**	91 ± 44	111 ± 76	0.126	0.306	0.062
**Vegetables**	73 ± 63	67 ± 53	0.874	0.603	<0.001 *
**Fruit juice**	44 ± 72	45 ± 90	0.764	0.350	0.031 *
**Sweetened beverages**	7 ± 12	89 ± 94	<0.001 *	0.301	0.067
**Candy**	12 ± 11	15 ± 16	0.122	0.441	0.006 *
**Ice cream**	6 ± 8	11 ± 15	0.156	0.295	0.072
**Bakery products**	13 ± 20	17 ± 16	0.069	0.102	0.542

^1^ Number of recorded days using 24 h dietary recalls: four days (*n* = 26, 68%), three days (*n* = 6, 16%), two days (*n* = 4, 11%), and one day (*n* = 2, 5%); ^2^ *p*-value is the difference between the intake between KidMeal-Q and 24 h dietary recalls calculated using the Wilcoxon Signed Ranks test; ^3^ Spearman rank order correlation; ^4^ *p*-value for the Spearman rank order correlation (rho). * Significant difference observed (*p* < 0.05).

**Table 3:** Mean intakes and correlations for selected nutrients estimated by KidMeal-Q and 24 h dietary recalls (*n* = 38).

	KidMeal-Q	24 h Dietary Recall	*p*-Value ^1^	*r* ^2^	*p*-Value ^3^
**Protein (E% ^4^)**	16 ± 2	16 ± 2	0.693	0.537	0.001 *
**Fat (E% ^4^)**	30 ± 3	31 ± 4	0.398	0.384	0.017 *
**Carbohydrates (E% ^4^)**	52 ± 4	52 ± 5	0.707	0.325	0.047 *
**Saturated fat E(% ^4^)**	13 ± 2	13 ± 2	0.877	0.279	0.090
**Monounsaturated fat (E% ^4^)**	11 ± 1	11 ± 2	0.145	0.282	0.087
**Polyunsaturated fat (E% ^4^)**	3.9 ± 0.6	3.9 ± 1.1	0.726	0.104	0.534
**Sucrose (E% ^4^)**	7 ± 2	11 ± 4	<0.001 *	0.459	0.004 *
**Fibre (g/MJ)**	3.0 ± 0.8	2.2 ± 0.6	<0.001 *	0.603	<0.001 *
**Vitamin A (ug/MJ)**	97 ± 30	91 ± 63	0.562	0.467	0.003 *
**Vitamin D (ug/MJ)**	0.78 ± 0.21	0.67 ± 0.36	0.051	0.426	0.008 *
**Vitamin E (ug/MJ)**	1.00 ± 0.15	0.95 ± 0.29	0.272	0.430	0.007 *
**Thiamin (mg/MJ)**	0.18 ± 0.03	0.17 ± 0.05	0.065	0.460	0.004 *
**Vitamin C (mg/MJ)**	13 ± 6	11 ± 7	0.115	0.610	<0.001 *
**Vitamin B12 (ug/MJ)**	0.65 ± 0.19	0.61 ± 0.25	0.364	0.439	0.006 *
**Calcium (mg/MJ)**	143 ± 40	113 ± 28	<0.001 *	0.491	0.002 *
**Iron (mg/MJ)**	1.5 ± 0.4	1.3 ± 0.5	0.069	0.486	0.002 *

^1^ *p*-value is the difference between the intake between KidMeal-Q and 24 h dietary recalls calculated using a paired samples *t*-test; ^2^ Pearson correlation coefficient; ^3^ *p*-value for the Pearson correlation coefficient (*r*); ^4^ Percent energy of total energy intake. * Significant difference observed (*p* < 0.05).

## Supplementary Materials

The following are available online at http://www.mdpi.com/2072-6643/9/1/66/s1.

## Abbreviations

The following abbreviations are used in this manuscript:

DLW	Doubly labelled water
EI	Energy intake
FFQ	Food frequency questionnaire
SD	Standard deviation
TEE	Total energy expenditure
