# Supplementary material for: Validation of an Online Food Frequency Questionnaire against Doubly Labelled Water and 24 h Dietary Recalls in Pre-School Children
Source: Nutrients. 2017 Jan 13;9(1):66. doi: 10.3390/nu9010066 (PMC5295110; doi:10.3390/nu9010066)
Supplement: Supplementary file 1 [file nutrients-09-00066-s001.docx]

Supplementary Materials: Validation of an Online Food Frequency Questionnaire against Doubly Labelled Water and 24 h Dietary Recalls in Pre-School Children

Christine Delisle Nyströ, Hanna Henriksson, Christina Alexandrou, Anna Bergström, Stephanie Bonn, Katarina Bälter and Marie Löf

KidMeal-Q:

Food habits:

Think about the following when you answer the questions:

- How your child has eaten in the past few months
- How your child usually eats both on weekdays and weekend days
- You only need to answer for the foods/drinks your child consumes
- The foods/drinks your child rarely or never consumes you can skip

How often does your child usually eat or drink something at the following meal times?

|  | **Everyday** | **Several Times Throughout the Week** | **Occasionally Throughout the Week** | **Seldom or Not at All** |
| --- | --- | --- | --- | --- |
| Breakfast |  |  |  |  |
| Lunch |  |  |  |  |
| Dinner |  |  |  |  |

How often does your child usually snack?

4 times a day or more

3 times a day or more

1–2 times per day

A few times per week

Seldom or never

Do not know/will not answer

For every beverage your child consumes at least once a month, choose in the list below how often your child usually consumes each of them.

Only fill in for the beverages your child has drunk in the last few months. You do not need to enter an answer if your child consumes a certain beverage rarely or not at all.

|  | **3 Times/Day or More** | **2 Times/Day** | **1 Time/Day** | **3–6 Times/Week** | **1–2 Times/Week** | **1–3 Times/Month** |
| --- | --- | --- | --- | --- | --- | --- |
| Water (tap or bottled), 1 glass |  |  |  |  |  |  |
| Milk or milk alternatives in a glass or bowl |  |  |  |  |  |  |
| Juice |  |  |  |  |  |  |
| Soda or sweetened beverages |  |  |  |  |  |  |
| Gruel |  |  |  |  |  |  |

You entered that your child drinks soda or sweetened beverages. Which beverage does your child consume the most?

Select all that are relevant.

Diet or calorie-free soda or sweetened beverages (i.e., beverages with artificial sweeteners)

Soda or sweetened beverages sweetened with sugar

Do not know/will not answer

For every food your child eats at least once a month, choose in the list below how often your child usually consumes each of them.

Only fill in for the foods your child usually eats.

|  | **3 Times/Day or More** | **2 Times/Day** | **1 Time/Day** | **3–6 Times/Week** | **1–2 Times/Week** | **1–3 Times/Month** |
| --- | --- | --- | --- | --- | --- | --- |
| Yogurt or yogurt drink |  |  |  |  |  |  |
| Cereal or muesli |  |  |  |  |  |  |
| Porridge |  |  |  |  |  |  |
| Fruit or berries |  |  |  |  |  |  |
| Jam or apple sauce |  |  |  |  |  |  |

You entered that your child eats yogurt.

Which of the following varieties of yogurt does your child usually eat?

Select all that are relevant.

Natural

With fruit, berries, or vanilla-flavored

Another variety of yogurt

Do not know/will not answer

You entered that your child eats cereal or muesli.

Which of the following varieties of cereal or muesli does your child usually eat?

Select all that are relevant.

Cornflakes or Special K

Muesli or high-fiber cereal (e.g., All Bran)

Cheerios or similar

Sweetened cereal (e.g., Frosted Flakes or Cocoa Puffs)

Another variety of cereal

Do not know/will not answer

You entered that your child eats porridge.

Which of the following varieties of porridge does your child usually eat?

Select all that are relevant.

Oatmeal

Cream of wheat

Children’s powder-based porridge

Another variety of porridge

Do not know/will not answer

You entered that your child eats fruits and berries.

Which of the following varieties of fruit and berries does your child usually eat?

Select all that are relevant.

Banana

Apple or pear

Citrus fruits (e.g., oranges or satsumas)

Berries (e.g., blueberries, raspberries, or strawberries)

Kiwi

Grapes

Another type of fruit

Do not know/will not answer

For every food your child eats at least once a month, choose in the list below how often your child usually consumes each of them.

Only fill in for the foods your child usually eats.

|  | **3 Times/Day or More** | **2 Times/Day** | **1 Time/Day** | **3–6 Times/Week** | **1–2 Times/Week** | **1–3 Times/Month** |
| --- | --- | --- | --- | --- | --- | --- |
| White bread |  |  |  |  |  |  |
| Whole wheat bread |  |  |  |  |  |  |
| Hard bread |  |  |  |  |  |  |
| Rice cakes or digestive biscuits |  |  |  |  |  |  |
| Sandwich toppings (e.g., cheese or ham) |  |  |  |  |  |  |
| Eggs |  |  |  |  |  |  |

You entered that your child eats bread.

How many slices of bread does your child usually eat at each meal?

Less than 1 slice

1–2 slices

3–4 slices

5–6 slices

7 slices or more

Do not know/will not answer

What type of butter or margarine does your child usually have on their sandwiches?

Margarine (e.g., Becel)

Butter

Another type of butter or margarine

Does not use butter or margarine

Do not know/will not answer

Which of the following toppings does your child eat most often?

Select all that are relevant.

Cheese

Deli meat (e.g., ham or salami)

Marmalade

Liverwurst

Caviar

Cucumber

Tomato or peppers

Another type of topping

Do not know/will not answer

Cooked food

Think about the following when you answer the questions:

- How your child has eaten in the past few months
- How your child usually eats both on weekdays and weekend days
- You only need to answer for the dishes your child eats
- The dishes your child rarely or never consumes you can skip

How much meat, fish, or meat alternative does your child usually eat?

Select the alternative that fits best your child’s food habits.


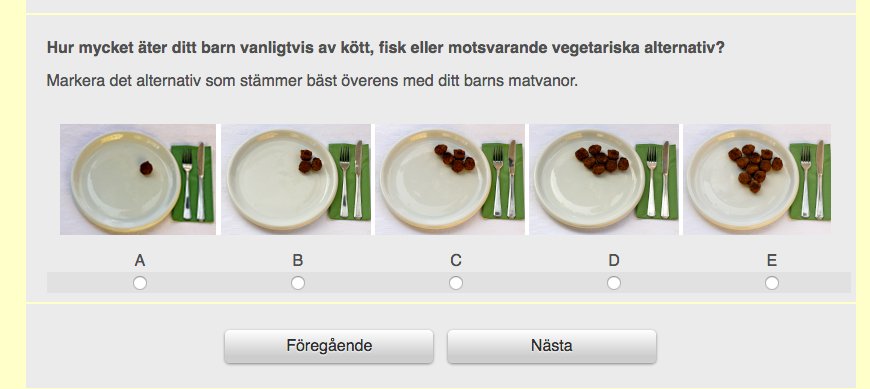


How much potatoes, rice, pasta, or something similar does your child usually eat?

Select the alternative that fits best your child’s food habits.


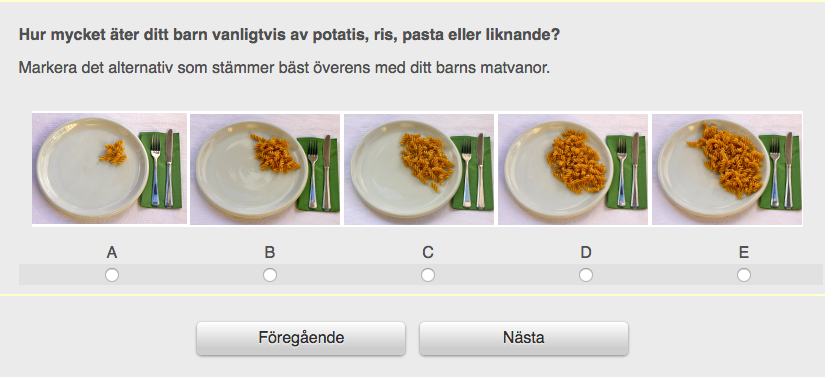


How much raw or cooked vegetables does your child usually eat?

Select the alternative that fits best your child’s food habits.


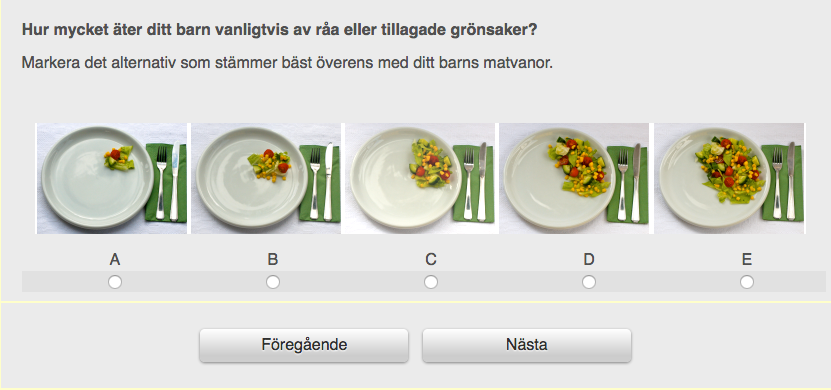


For every meat dish your child eats at least once a month, choose in the list below how often your child usually consumes each of them.

Only fill in for the dishes your child usually eats.

|  | **7 Times/Week or More** | **5–6 Times/Week** | **3–4 Times/Week** | **1–2 Times/Week** | **1–3 Times/Month** |
| --- | --- | --- | --- | --- | --- |
| Hamburger (including the bun) or Tex-mex (e.g., tacos) |  |  |  |  |  |
| Minced meat dishes (e.g., meat sauce, lasagna, or meatballs) |  |  |  |  |  |
| Lasagna, moussaka, or tortellini with meat |  |  |  |  |  |
| Chicken or turkey (e.g., grilled or in a wok or stew) |  |  |  |  |  |
| Meat dishes (e.g., fried or in a stew or wok) |  |  |  |  |  |
| Sausage dishes (e.g., fried, boiled, or in a stew) |  |  |  |  |  |
| Blood pudding |  |  |  |  |  |

For every food/dish your child eats at least once a month, choose in the list below how often your child usually consumes each of them.

Only fill in for the dishes your child usually eats.

|  | **7 Times/Week or More** | **5–6 Times/Week** | **3–4 Times/Week** | **1–2 Times/Week** | **1–3 Times/Month** |
| --- | --- | --- | --- | --- | --- |
| Fish or shellfish |  |  |  |  |  |
| Vegetarian dishes (e.g., lentil stew, bean burgers, soy sausages, or Quorn) |  |  |  |  |  |
| Pizza, pie/quiche, or pierogis |  |  |  |  |  |
| Pancakes or waffles |  |  |  |  |  |
| Soup (e.g., tomato, pea, or goulash) |  |  |  |  |  |

You entered that your child eats fish or shellfish.

Which of the following dishes does your child usually eat?

Select all that are relevant.

Fish sticks

Cod, haddock, or another type of white fish (not fish sticks)

Salmon, sushi, mackerel, or pickled herring

Tuna

Shellfish (e.g., shrimp or mussels)

Another type of fish

Do not know/will not answer

You entered that your child eats vegetarian protein-rich dishes.

Which of the following dishes does your child usually eat?

Select all that are relevant.

Beans, lentils, chickpeas, (e.g., lentil stew, bean burgers, or falafel)

Soy meat, soy sausages, or tofu

Quorn or Quorn stew

Root vegetable dishes (e.g., beets, parsnips, or rutabaga)

Do not know/will not answer

For every food your child eats at least once a month, choose in the list below how often your child usually consumes each of them.

Only fill in for the dishes your child usually eats.

|  | **7 Times/Week or More** | **5–6 Times/Week** | **3–4 Times/Week** | **1–2 Times/Week** | **1–3 Times/Month** |
| --- | --- | --- | --- | --- | --- |
| Vegetables, raw or cooked |  |  |  |  |  |
| Cooked potatoes or mashed potatoes |  |  |  |  |  |
| French fries, hash browns, fried potatoes, or scalloped potatoes |  |  |  |  |  |
| Pasta or noodles |  |  |  |  |  |
| Rice |  |  |  |  |  |
| Bulgur, couscous, or quinoa |  |  |  |  |  |
| Condiments (e.g., ketchup or sauce) |  |  |  |  |  |

You entered that your child eats vegetables.

How many times per day does your child eat vegetables?

1 time

2 times

3 times or more

Do not know/will not answer

You entered that your child eats vegetables.

Which of the following vegetables does your child eat the most often?

Select all that are relevant.

Tomato or peppers

Cucumber, lettuce, or zucchini

Carrots

Onion

Corn

Avocado

Cauliflower or cabbage (e.g., coleslaw)

Broccoli

Spinach

Green peas

Beans, lentils, or chickpeas

Parsnips, rutabaga, or celery root

Pickled vegetables

Another vegetable

Do not know/will not answer

You entered that your child eats condiments (e.g., ketchup or sauce) on their food.

Which of the following condiments does your child eat the most often?

Select all that are relevant.

Ketchup, tomato sauce, or tomato salsa

Vinaigrette (oil and vinegar)

Cream sauce or crème fraiche/sour cream sauce

Béarnaise sauce or mayonnaise-based sauce

Another sauce

Do not know/will not answer

For snacks and candy your child eats at least once a month, choose in the list below how often your child usually consumes each of them.

Only fill in for the foods your child usually eats.

|  | **3 Times/Day or More** | **2 Times/Day** | **1 Time/Day** | **3–6 Times/Week** | **1–2 Times/Week** | **1–3 Times/Month** |
| --- | --- | --- | --- | --- | --- | --- |
| Buns or cookies |  |  |  |  |  |  |
| Chocolate or candy |  |  |  |  |  |  |
| Raisins |  |  |  |  |  |  |
| Ice cream |  |  |  |  |  |  |
| Nuts |  |  |  |  |  |  |
| Snacks (e.g., chips, popcorn, or cheezies) |  |  |  |  |  |  |

How much chocolate or candy does your child usually eat every time?

One chocolate bar is equivalent to 100 grams and a large chocolate bar is equivalent to 200 grams.

One bag of candy (e.g., gummy bears or jelly beans) is equivalent to 150 grams and a small box is equivalent to 25 grams.

A single candy or chocolate square

50 grams

100 grams

Do not know/will not answer

You entered that your child eats ice cream.

How much ice cream does your child usually eat on every occasion?

1 scoop of ice cream is equivalent to 50 grams or approximately 1 dL.

Less than 1 scoop

1 scoop

2 scoops

3 scoops or more

Do not know/will not answer

You entered that your child eats chips, popcorn, or cheezies.

How much chips, popcorn, or cheezies does your child usually eat on every occasion?

1 normal bag of chips weighs 200 grams.

A single chip, piece of popcorn, or cheezie

50 grams

100 grams or more

Do not know/will not answer

Does your child eat products with probiotics, i.e., “good bacteria” that are added to yogurts or fruit juices for example?

Yes

No

Do not know/will not answer

How often does your child eat products with probiotics, i.e., “good bacteria”?

Everyday

Several times per week

A few times per week

Rarely or never

Do not know/will not answer

Does your child usually consume vitamins, minerals, or another supplement?

Yes, regularly

Yes, sometimes

No

Do not know/will not answer

For the supplements your child has consumed in the past few months, choose in the list below how often your child usually consumes each of them.

Only fill in for the supplements your child usually consumes.

|  | **Everyday** | **A Few Times per Week** | **A Few Times per Month** | **Periodically** |
| --- | --- | --- | --- | --- |
| Multivitamin or mineral |  |  |  |  |
| Vitamin A |  |  |  |  |
| B vitamins |  |  |  |  |
| Vitamin C |  |  |  |  |
| Vitamin D |  |  |  |  |
| Vitamin E |  |  |  |  |
| Folic acid |  |  |  |  |
| Iron |  |  |  |  |
| Calcium |  |  |  |  |
| Fish oil (omega-3) |  |  |  |  |
| Probiotics |  |  |  |  |
